# Postoperative mortality analysis on nationwide data from diagnosis procedure combination database in Japan

**DOI:** 10.1371/journal.pone.0286264

**Published:** 2023-06-08

**Authors:** Susumu Kunisawa

**Affiliations:** Department of Healthcare Economics and Quality Management, Graduate School of Medicine, Kyoto University, Kyoto City, Japan; Research Institute of Tuberculosis, JAPAN

## Abstract

**Introduction:**

The present study aimed to investigate the postoperative mortality due to all surgeries at the prefectural level using a nationwide diagnosis procedure combination (DPC) database in Japan and to evaluate the data according to temporal changes and regional differences.

**Methods:**

Data were provided in accordance with the guidelines indicated on the Ministry of Health, Labor and Welfare, Japan. The number of cases and in-hospital mortality were calculated for each representative surgery for each hospitalization according to fiscal year of discharge from 2011 to 2018 and according to prefecture. Values of ≥10 in each aggregated data cell were presented.

**Results and discussion:**

The aggregated result data contain 474,154 records, with about 2,000 different surgical codes. More than 10 mortalities were recorded in only 16,890 data cells, which can be used in the mortality analysis. In the analyses of artificial head insertion, cerebral aneurysm neck clipping, coronary artery and aortic bypass grafting, and tracheotomy, regional differences and a declining trend were observed in some categories.

**Conclusion:**

In addition to considering categories that can be used in the analysis, careful consideration must be given to the inclusion of background context such as the quality of care.

## 1. Introduction

Improving quality of care is one of the greatest concerns in the medical community, and reducing postoperative complications deserves attention. This is a common concern worldwide; however, there have been reports of relatively few perioperative complications in Japan compared to other countries [1, 2]. In Japan, various case registries have been conducted, verified, and reviewed, mainly at academic conferences [3, 4]. Currently, a large volume of case data is available the Japanese surgical field, primarily through the National Clinical Database (NCD), and these data are being used for validation and feedback to facilities. However, data that can be obtained from such case registries is limited to the data registered in them. Although some surgical procedures have fairly high registration rates [5], registration must be a proactive approach; moreover, registration omissions can still occur. In addition, databases, such as the NCD (https://www.ncd.or.jp/press/), do not necessarily include data from all surgical areas, although they are being used in major surgeries such as gastroenterological surgery, cardiovascular surgery, respiratory surgery, and pediatric surgery [6–8]. This can be a limiting factor for studies based on any case registries that are conducted not only in Japan but also in other countries.

Exhaustive analyses using reimbursement or administrative data that are not registry databases are readily being conducted. In Japan, along with the evolution of information technology, the diagnosis procedure combination (DPC) database that included data obtained via the DPC per-diem payment system (DPC/PDPS), which was started in 2003, was created in tandem with the accumulation and utilization of DPC data. DPC data is prepared as a discharge summary called “Form 1” in addition to documenting—at the voucher level—the medical treatment activities performed during treatment, despite being a comprehensive payment system. Initially, these data were defined according to the DPC/PDPS system; however, because medical institutions that have not adopted DPC/PDPS were also approached for creating the DPC database, this database now includes data created and submitted to the Ministry of Health, Labor and Welfare (MHLW) by the majority of hospitals (5,315 hospitals in 2020; https://www.mhlw.go.jp/stf/shingi2/0000196043_00005.html).

The DPC database, containing data on most hospitalizations related to insured care, has been applied in practical management and clinical research analyses from various perspectives. This database has been very useful for research across organizations because it contains government-defined data. Notably, many intrahospital analyses and cross-sectional or interhospital analyses within multiple organizations or groups have been conducted [9–13]. Moreover, since 2017, data collected by the MHLW could be used for analyses. Previously, a study successfully calculated process indicators using this database [14] and distinctly showed the temporal changes and regional differences in Japan.

Postoperative mortality can be considered one of the quality indicators. Although mortality is associated with various parameters such as the technical quality, case-mix differences, and differential indication strategies, the mortality rates in Japan are often lower than those in other countries [1, 2]. However, such results are obtained from the analysis of data from specific hospitals or from a data registry. Therefore, the present study was designed to explore regional differences and temporal changes in postoperative mortality for any surgery, with quality of care as an outcome measure using a comprehensive nationwide database from Japan. Instead of examining details such as the causes of regional differences in specific surgeries, the present study fundamentally calculated and provided these indicators, thereby providing further insights and facilitating detailed and specific studies in the future. In this analysis, some stipulations for data usage for cross-disciplinary indicators are presented and several items that should be considered by future investigators are reported.

## 2. Material and methods

Data were obtained in accordance with the guidelines and applications indicated on the MHLW website. The number of cases and in-hospital mortality were calculated for each representative surgery for each hospitalization according to the fiscal year of discharge from 2011 to 2018 and according to prefecture; data were provided as an aggregated result from the MHLW. The “Form 1” of DPC data facilitates the recording of a maximum of five surgeries, and the primary surgery or the one with the highest surgical fee is documented as the first surgery. Surgical information recorded included the surgery name and surgical code (K-code). In this study, data were analyzed and presented based on K-codes. As this coding is specifically used only in the Japanese healthcare system, the used coding system has been provided in the S1 Data. As the surgery name causes some notational inconsistencies, data were tabulated according to the surgical code; the standard master surgery names for those codes in Japanese are available at the Various Information of Medical Fee website (http://shinryohoshu.mhlw.go.jp/shinryohoshu/), and the referable translation is available at the Japan Surgical Society—Web Glossary of Surgical Terms (http://yougosyu.jssoc.or.jp/). Data that can be obtained as an aggregate has been limited by the rules of MHLW to a value of ≥10 in each cell for anonymity. If the obtained value was <10, it was removed and nullified, but was indicated as “-” in this report. Not all of these obtained aggregated data are disclosed in this report in accordance with the guidelines of MHLW; in addition, the number was removed and indicated as “*-” when the difference between the number of cases and in-hospital mortality was <10 (in S1 Data). In the present study, postoperative in-hospital mortality was defined and calculated as the number of in-hospital mortality divided by the number of postoperative case discharges. Therefore, patients who were discharged alive once and died during events such as readmission were not included in the mortality data.

The number of DPC-participating hospitals is increasing annually. As mentioned above, the submission of DPC data has been expanded to non-DPC hospitals in recent years, with each progressive year showing an increase in the number of hospitals submitting DPC data. Consequently, more cases are being covered by this database; thus, the case volume has increased.

All surgical data obtained in the present study is presented as S1 Data. Across the entire spectrum, each result has its own importance and requires interpretation, but this report selected the most distinctive ones for consideration. Although this selection was arbitrarily made by the author, the inclusion criteria were as follows: those with relatively high mortality rates, those with visible temporal changes or regional differences, those requiring interpretation, or those requiring caution in future data analysis.

The present study was approved by Kyoto University Graduate School and Faculty of Medicine, Ethics Committee. The need for obtaining individual informed consent was waived for this study as that data were anonymized and provided by the MHLW in accordance with the “Strategy for the Revitalization of Japan” 2016 (approved by the Cabinet on June 2, 2016). The data presented in this study were independently requested and analyzed by the author and are different from the data such as statistics prepared and published by the MHLW Japan.

## 3. Results

All data obtained in this study are presented as S1 Data, except for the unknown operation code data of 24 records. The dataset contained 474,154 records; for each year, the dataset comprised 1,986–2,496 different codes. Records with 0 cases were not included in the dataset; 221,866 records contained >10 cases. Only 16,890 records exhibited >10 mortalities, and 186 records masked the methods. With 56 surgical codes, the mortality rates for all prefectures during a fiscal year were comparable, and with 1,063 surgical codes, the mortality rates during a fiscal year were comparable among prefectures.

### 3.1. Artificial head insertion for the shoulder or hip (K0811)

**Fig 1:**
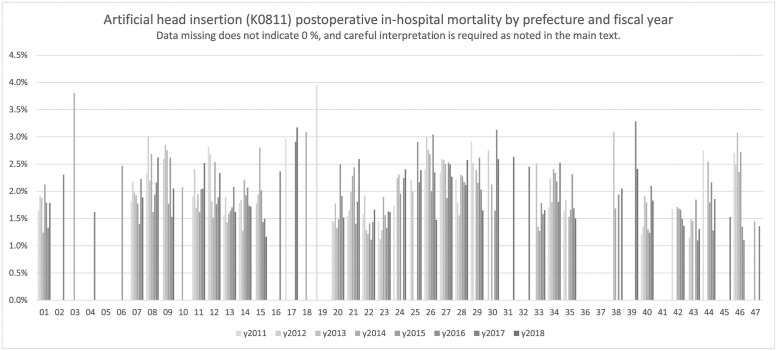
Artificial head insertion for the shoulder or hip (K0811). Artificial head insertion (K0811) postoperative in-hospital mortality by prefecture and fiscal year.

Fig 1 depicts the postoperative in-hospital mortality of artificial head insertion for the shoulder or hip in orthopedic surgery, and Table 1 shows the detail results for those cases. Surgeries coded with K0811 were artificial head insertions for the shoulder or hip; therefore, the cases were mixed. Moreover, postoperative in-hospital mortality rate was approximately 1%–3% and appeared to slightly vary regionally. However, there were many regions where numerical aggregation was impossible because of low postoperative in-hospital mortalities in each area.

**Table 1:** Artificial head insertion (K0811): Case number and outcomes of postoperative in-hospital mortality.

Fiscal year	2011			2012			2013			2014			2015			2016			2017			2018		
Prefecture	Case	Outcome		Case	Outcome		Case	Outcome		Case	Outcome		Case	Outcome		Case	Outcome		Case	Outcome		Case	Outcome	
01	1221	20	1.6%	1358	26	1.9%	1436	27	1.9%	1692	21	1.2%	1978	42	2.1%	2119	38	1.8%	2254	30	1.3%	2406	43	1.8%
02	286	-	-	321	-	-	286	-	-	342	-	-	378	-	-	432	-	-	477	11	2.3%	500	-	-
03	319	-	-	334	-	-	329	-	-	368	14	3.8%	399	-	-	364	-	-	453	-	-	528	-	-
04	555	-	-	536	-	-	486	-	-	619	-	-	613	-	-	744	-	-	741	12	1.6%	816	-	-
05	306	-	-	265	-	-	290	-	-	312	-	-	357	-	-	352	-	-	385	-	-	371	-	-
06	390	-	-	375	-	-	363	-	-	379	-	-	405	10	2.5%	378	-	-	452	-	-	435	-	-
07	551	10	1.8%	550	12	2.2%	556	11	2.0%	626	12	1.9%	676	12	1.8%	715	10	1.4%	808	18	2.2%	849	16	1.9%
08	775	18	2.3%	796	24	3.0%	683	15	2.2%	895	24	2.7%	928	15	1.6%	1032	20	1.9%	1204	26	2.2%	1221	32	2.6%
09	366	-	-	424	11	2.6%	490	14	2.9%	581	16	2.8%	677	12	1.8%	687	18	2.6%	784	12	1.5%	877	18	2.1%
10	411	-	-	499	-	-	498	-	-	579	12	2.1%	664	-	-	702	-	-	763	-	-	691	-	-
11	1420	27	1.9%	1535	37	2.4%	1648	28	1.7%	1954	38	1.9%	2289	37	1.6%	2651	54	2.0%	2834	58	2.0%	3094	78	2.5%
12	1173	33	2.8%	1269	34	2.7%	1321	24	1.8%	1650	25	1.5%	1855	47	2.5%	2267	40	1.8%	2334	44	1.9%	2526	59	2.3%
13	3709	58	1.6%	3843	73	1.9%	4198	60	1.4%	4720	75	1.6%	5049	83	1.6%	5341	91	1.7%	5957	124	2.1%	6230	101	1.6%
14	2860	51	1.8%	3047	56	1.8%	3358	43	1.3%	3721	82	2.2%	4050	78	1.9%	4300	89	2.1%	4547	79	1.7%	4706	81	1.7%
15	547	-	-	619	11	1.8%	720	14	1.9%	785	22	2.8%	990	20	2.0%	975	14	1.4%	1136	17	1.5%	1117	13	1.2%
16	407	-	-	412	-	-	459	-	-	433	-	-	564	-	-	540	-	-	549	13	2.4%	516	-	-
17	370	11	3.0%	350	-	-	331	-	-	404	-	-	446	-	-	434	-	-	481	14	2.9%	504	16	3.2%
18	297	-	-	339	-	-	342	-	-	356	11	3.1%	315	-	-	327	-	-	344	-	-	381	-	-
19	253	10	4.0%	267	-	-	275	-	-	282	-	-	306	-	-	365	-	-	425	-	-	469	-	-
20	686	10	1.5%	763	11	1.4%	788	14	1.8%	904	12	1.3%	943	14	1.5%	960	24	2.5%	1047	20	1.9%	1056	16	1.5%
21	905	14	1.5%	909	15	1.7%	857	17	2.0%	1050	24	2.3%	1107	27	2.4%	1068	15	1.4%	1217	22	1.8%	1235	32	2.6%
22	1522	24	1.6%	1509	29	1.9%	1555	20	1.3%	1727	21	1.2%	1774	25	1.4%	1891	21	1.1%	2022	29	1.4%	1985	33	1.7%
23	1796	26	1.4%	2052	23	1.1%	2254	29	1.3%	2424	46	1.9%	2758	43	1.6%	2872	38	1.3%	3006	49	1.6%	3024	49	1.6%
24	693	12	1.7%	704	-	-	757	17	2.2%	823	19	2.3%	923	18	2.0%	975	-	-	1160	26	2.2%	1123	27	2.4%
25	462	-	-	453	10	2.2%	501	10	2.0%	526	-	-	598	-	-	654	19	2.9%	692	15	2.2%	670	16	2.4%
26	790	19	2.4%	965	29	3.0%	978	27	2.8%	1082	29	2.7%	1198	24	2.0%	1215	37	3.0%	1364	32	2.3%	1423	21	1.5%
27	2986	70	2.3%	3280	85	2.6%	3376	87	2.6%	3961	99	2.5%	4426	83	1.9%	4593	116	2.5%	4973	124	2.5%	5376	122	2.3%
28	1764	39	2.2%	1910	34	1.8%	1991	31	1.6%	2302	53	2.3%	2815	64	2.3%	2996	65	2.2%	3119	66	2.1%	3225	83	2.6%
29	618	18	2.9%	596	15	2.5%	685	-	-	628	15	2.4%	742	16	2.2%	764	20	2.6%	837	17	2.0%	1031	17	1.6%
30	489	-	-	545	15	2.8%	523	-	-	612	13	2.1%	610	-	-	668	11	1.6%	767	24	3.1%	734	19	2.6%
31	234	-	-	305	-	-	266	-	-	295	-	-	403	-	-	382	-	-	370	-	-	418	11	2.6%
32	240	-	-	313	-	-	293	-	-	349	-	-	371	-	-	393	-	-	465	-	-	408	10	2.5%
33	681	-	-	805	-	-	796	20	2.5%	896	12	1.3%	1021	13	1.3%	1064	19	1.8%	1202	19	1.6%	1147	19	1.7%
34	998	17	1.7%	1076	24	2.2%	1112	20	1.8%	1246	30	2.4%	1368	32	2.3%	1511	33	2.2%	1606	29	1.8%	1743	44	2.5%
35	612	10	1.6%	653	12	1.8%	680	-	-	782	12	1.5%	843	14	1.7%	865	20	2.3%	947	16	1.7%	997	15	1.5%
36	322	-	-	411	-	-	405	-	-	524	-	-	554	-	-	621	-	-	587	-	-	592	-	-
37	390	-	-	411	-	-	430	-	-	432	-	-	463	-	-	466	-	-	559	-	-	557	-	-
38	355	-	-	396	-	-	452	14	3.1%	593	10	1.7%	687	-	-	722	14	1.9%	794	-	-	878	18	2.1%
39	329	-	-	353	-	-	356	-	-	369	-	-	388	-	-	369	-	-	457	15	3.3%	456	11	2.4%
40	1991	24	1.2%	2071	28	1.4%	2136	41	1.9%	2344	42	1.8%	2537	33	1.3%	2750	34	1.2%	2906	61	2.1%	2953	54	1.8%
41	320	-	-	334	-	-	341	-	-	389	-	-	485	-	-	481	-	-	498	-	-	473	-	-
42	657	11	1.7%	674	-	-	701	-	-	818	14	1.7%	833	14	1.7%	902	15	1.7%	1002	15	1.5%	1026	14	1.4%
43	935	-	-	959	11	1.1%	938	14	1.5%	1031	15	1.5%	1046	-	-	1192	22	1.8%	1184	13	1.1%	1150	15	1.3%
44	400	11	2.8%	521	-	-	523	-	-	551	14	2.5%	723	13	1.8%	693	15	2.2%	782	10	1.3%	755	14	1.9%
45	392	-	-	396	-	-	413	-	-	561	-	-	596	-	-	627	-	-	690	-	-	655	10	1.5%
46	519	14	2.7%	560	14	2.5%	488	15	3.1%	637	15	2.4%	735	20	2.7%	816	11	1.3%	903	10	1.1%	960	-	-
47	624	-	-	630	-	-	683	-	-	692	10	1.4%	712	-	-	759	-	-	736	10	1.4%	743	-	-

“-” indicates the value of <10.

### 3.2. Cerebral aneurysm neck clipping (1 location; K1771)

**Fig 2:**
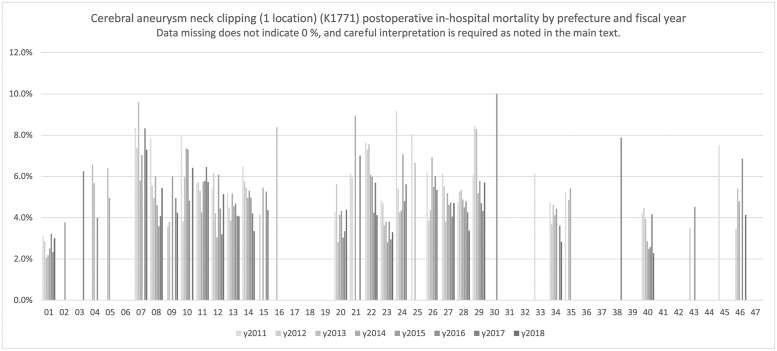
Cerebral aneurysm neck clipping (one location) (K1771). Cerebral aneurysm neck clipping (1 location) (K1771) postoperative in-hospital mortality by prefecture and fiscal year.

Fig 2 depicts the postoperative in-hospital mortality of 1 location cerebral aneurysm neck clipping, and Table 2 shows the results for those cases. The code K1771 and K1772 were used to indicate clipping of one location and multiple locations, respectively. A decreasing trend over time can be observed in 14 (Miyagi Prefecture), 22 (Shizuoka Prefecture), and 23 (Aichi Prefecture) records.

**Table 2:** Cerebral aneurysm neck clipping (1 location; K1771): Case number and outcomes of postoperative in-hospital mortality.

Fiscal year	2011			2012			2013			2014			2015			2016			2017			2018		
Prefecture	Case	Outcome		Case	Outcome		Case	Outcome		Case	Outcome		Case	Outcome		Case	Outcome		Case	Outcome		Case	Outcome	
01	1225	38	3.1%	1299	37	2.8%	1264	26	2.1%	1288	28	2.2%	1314	33	2.5%	1209	39	3.2%	1158	27	2.3%	1164	35	3.0%
02	272	-	-	258	-	-	241	-	-	232	-	-	265	10	3.8%	226	-	-	209	-	-	171	-	-
03	257	-	-	259	-	-	241	-	-	202	-	-	182	-	-	176	-	-	176	11	6.3%	163	-	-
04	197	-	-	194	-	-	152	10	6.6%	194	11	5.7%	274	-	-	275	11	4.0%	264	-	-	250	-	-
05	136	-	-	117	-	-	172	11	6.4%	202	10	5.0%	178	-	-	167	-	-	165	-	-	145	-	-
06	175	-	-	182	-	-	156	-	-	179	-	-	187	-	-	157	-	-	162	-	-	135	-	-
07	263	22	8.4%	230	17	7.4%	219	21	9.6%	207	12	5.8%	185	13	7.0%	194	-	-	204	17	8.3%	192	14	7.3%
08	332	26	7.8%	433	24	5.5%	403	20	5.0%	433	26	6.0%	456	21	4.6%	419	15	3.6%	367	15	4.1%	405	22	5.4%
09	244	-	-	281	10	3.6%	290	11	3.8%	265	-	-	267	16	6.0%	250	-	-	263	13	4.9%	283	12	4.2%
10	252	20	7.9%	262	10	3.8%	218	13	6.0%	272	20	7.4%	260	19	7.3%	228	11	4.8%	210	-	-	218	14	6.4%
11	634	36	5.7%	683	39	5.7%	660	35	5.3%	705	30	4.3%	645	37	5.7%	726	42	5.8%	666	43	6.5%	698	40	5.7%
12	572	31	5.4%	585	36	6.2%	592	25	4.2%	590	18	3.1%	543	33	6.1%	540	24	4.4%	563	18	3.2%	564	29	5.1%
13	1626	84	5.2%	1646	74	4.5%	1636	63	3.9%	1527	79	5.2%	1538	70	4.6%	1406	66	4.7%	1394	57	4.1%	1352	55	4.1%
14	1036	67	6.5%	989	57	5.8%	880	48	5.5%	1007	50	5.0%	962	51	5.3%	986	49	5.0%	999	42	4.2%	957	32	3.3%
15	219	-	-	241	10	4.1%	224	-	-	220	12	5.5%	198	-	-	228	12	5.3%	229	10	4.4%	202	-	-
16	168	-	-	131	-	-	155	13	8.4%	139	-	-	127	-	-	123	-	-	124	-	-	137	-	-
17	130	-	-	114	-	-	112	-	-	104	-	-	105	-	-	104	-	-	106	-	-	77	-	-
18	94	-	-	92	-	-	93	-	-	91	-	-	71	-	-	72	-	-	59	-	-	65	-	-
19	75	-	-	76	-	-	96	-	-	80	-	-	83	-	-	82	-	-	68	-	-	74	-	-
20	375	16	4.3%	445	25	5.6%	427	12	2.8%	435	18	4.1%	392	17	4.3%	362	11	3.0%	329	11	3.3%	342	15	4.4%
21	212	13	6.1%	235	14	6.0%	210	-	-	179	16	8.9%	183	-	-	155	-	-	157	11	7.0%	196	-	-
22	589	45	7.6%	548	40	7.3%	503	38	7.6%	494	30	6.1%	501	30	6.0%	471	20	4.2%	474	27	5.7%	437	18	4.1%
23	889	43	4.8%	852	40	4.7%	854	31	3.6%	840	32	3.8%	891	25	2.8%	816	31	3.8%	847	25	3.0%	790	26	3.3%
24	218	20	9.2%	241	13	5.4%	235	10	4.3%	230	10	4.3%	184	13	7.1%	208	10	4.8%	196	11	5.6%	199	-	-
25	149	12	8.1%	127	-	-	150	10	6.7%	131	-	-	130	-	-	126	-	-	127	-	-	124	-	-
26	258	16	6.2%	259	10	3.9%	252	11	4.4%	231	16	6.9%	237	13	5.5%	216	13	6.0%	206	11	5.3%	152	-	-
27	1221	75	6.1%	1249	69	5.5%	1205	46	3.8%	1156	60	5.2%	1081	50	4.6%	1057	50	4.7%	1012	41	4.1%	870	41	4.7%
28	733	35	4.8%	780	41	5.3%	825	44	5.3%	801	39	4.9%	733	33	4.5%	751	36	4.8%	704	30	4.3%	682	23	3.4%
29	198	12	6.1%	202	17	8.4%	181	15	8.3%	212	11	5.2%	225	13	5.8%	234	11	4.7%	231	10	4.3%	228	13	5.7%
30	120	-	-	98	-	-	117	-	-	116	-	-	96	-	-	100	10	10.0%	70	-	-	76	-	-
31	97	-	-	103	-	-	81	-	-	70	-	-	76	-	-	52	-	-	74	-	-	59	-	-
32	93	-	-	83	-	-	93	-	-	83	-	-	84	-	-	74	-	-	75	-	-	73	-	-
33	244	15	6.1%	242	-	-	259	-	-	222	-	-	208	-	-	207	-	-	175	-	-	171	-	-
34	339	16	4.7%	299	11	3.7%	324	15	4.6%	291	12	4.1%	293	13	4.4%	291	-	-	305	11	3.6%	353	10	2.8%
35	191	10	5.2%	172	-	-	206	10	4.9%	203	11	5.4%	193	-	-	189	-	-	153	-	-	142	-	-
36	105	-	-	93	-	-	103	-	-	93	-	-	70	-	-	60	-	-	61	-	-	73	-	-
37	84	-	-	83	-	-	84	-	-	84	-	-	108	-	-	74	-	-	66	-	-	68	-	-
38	135	-	-	135	-	-	110	-	-	177	-	-	127	-	-	155	-	-	165	13	7.9%	125	-	-
39	120	-	-	96	-	-	120	-	-	82	-	-	83	-	-	96	-	-	104	-	-	94	-	-
40	803	34	4.2%	803	36	4.5%	711	28	3.9%	768	22	2.9%	723	18	2.5%	621	16	2.6%	624	26	4.2%	616	14	2.3%
41	102	-	-	99	-	-	104	-	-	136	-	-	118	-	-	118	-	-	115	-	-	102	-	-
42	229	-	-	207	-	-	192	-	-	178	-	-	185	-	-	198	-	-	195	-	-	202	-	-
43	343	-	-	316	11	3.5%	306	-	-	330	-	-	287	13	4.5%	265	-	-	293	-	-	242	-	-
44	106	-	-	115	-	-	89	-	-	73	-	-	101	-	-	118	-	-	90	-	-	85	-	-
45	146	11	7.5%	168	-	-	145	-	-	108	-	-	151	-	-	170	-	-	151	-	-	143	-	-
46	301	-	-	290	10	3.4%	296	16	5.4%	292	14	4.8%	290	-	-	248	17	6.9%	242	-	-	242	10	4.1%
47	144	-	-	182	-	-	124	-	-	121	-	-	127	-	-	130	-	-	151	-	-	134	-	-

“-” indicates the value of <10.

### 3.3. Coronary artery and aortic bypass grafting (K5521, K5522, K552-21, and K552-22)

**Fig 3:**
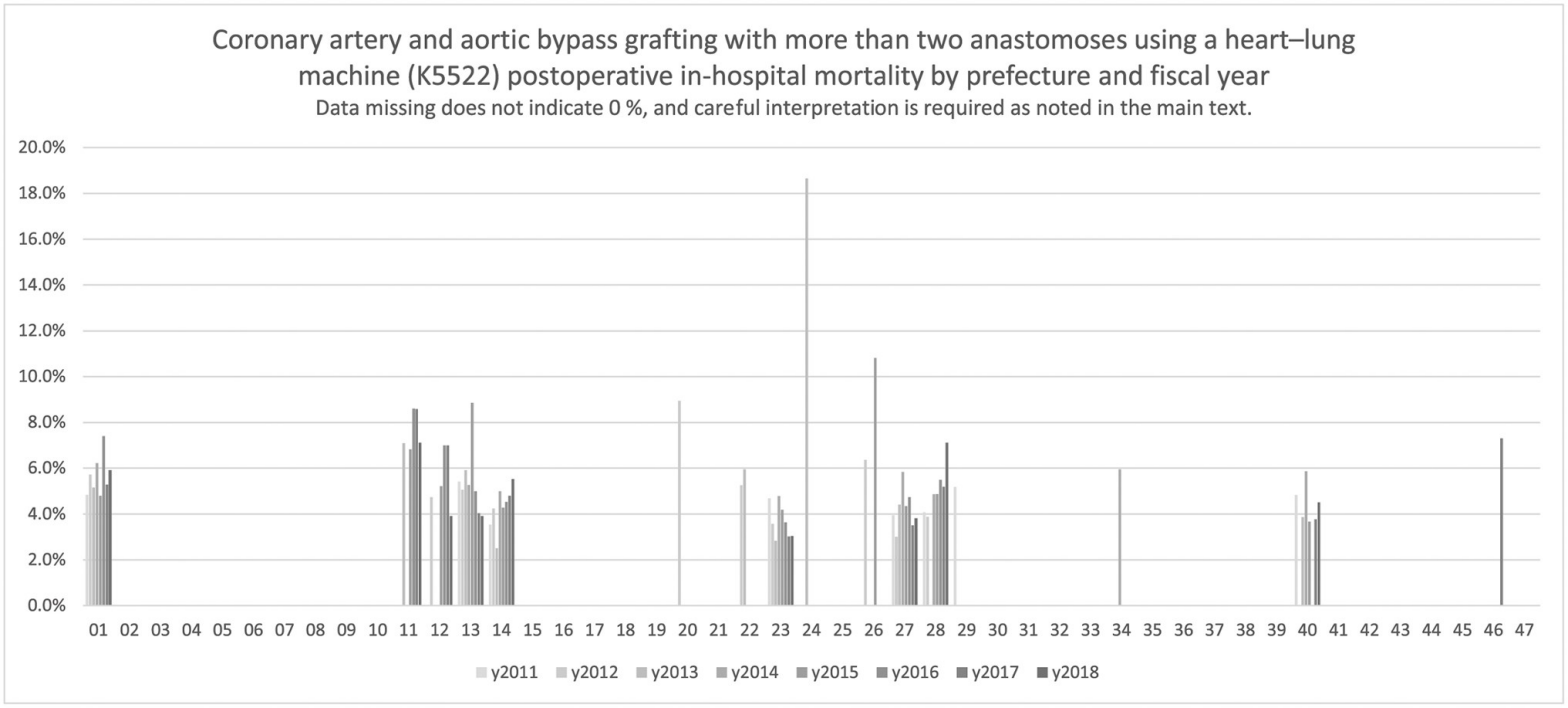
Coronary artery and aortic bypass grafting with more than two anastomoses using a heart–lung machine (K5522). Coronary artery and aortic bypass grafting with more than two anastomoses using a heart–lung machine (K5522) postoperative in-hospital mortality by prefecture and fiscal year.

Fig 3 depicts the postoperative in-hospital mortality in coronary artery and aortic bypass grafting of >2 anastomoses (K5522, Table 4), and Tables 3–6 show the results for coronary artery and aortic bypass grafting with 1 anastomosis using a heart–lung machine (K5521), >2 anastomoses using a heart–lung machine (K5522), with 1 anastomosis without a heart–lung machine (K552-21), and >2 anastomoses without a heart–lung machine (K552-22). Data shown in Table 4 included relatively more numerical results; for data shown the remaining tables, numerical results were available for the denominator, and the number of numerators in several regions was too small to be tabulated. Although only a few areas can be depicted as a result of the tally, regional differences were noted. As mentioned above, coronary artery bypass surgery was classified into four reimbursement categories. These could be considered different surgeries; however, the numerical value of outcomes for other coronary artery bypass grafting could not be depicted because the number of each outcome was small.

**Table 3:** Coronary artery and aortic bypass grafting with one anastomosis using a heart–lung machine (K5521): Case number and outcomes of postoperative in-hospital mortality.

Fiscal year	2011			2012			2013			2014			2015			2016			2017			2018		
Prefecture	Case	Outcome		Case	Outcome		Case	Outcome		Case	Outcome		Case	Outcome		Case	Outcome		Case	Outcome		Case	Outcome	
01	20	-	-	18	-	-	11	-	-	17	-	-	23	-	-	13	-	-	15	-	-	15	-	-
02	-	-	-										-	-	-	-	-	-	-	-	-			
03							-	-	-				-	-	-	-	-	-	-	-	-	-	-	-
04	-	-	-	-	-	-	13	-	-	11	-	-	-	-	-	-	-	-	-	-	-	16	-	-
05	-	-	-										-	-	-	-	-	-	-	-	-	-	-	-
06	-	-	-				-	-	-	-	-	-	-	-	-	-	-	-				-	-	-
07	-	-	-	-	-	-	-	-	-				-	-	-	-	-	-	-	-	-	-	-	-
08	-	-	-	-	-	-	-	-	-	-	-	-	-	-	-	-	-	-	-	-	-	-	-	-
09	-	-	-	-	-	-	-	-	-	-	-	-	-	-	-	-	-	-	-	-	-	-	-	-
10	-	-	-	-	-	-	-	-	-	-	-	-	-	-	-	-	-	-	-	-	-	-	-	-
11	19	-	-	-	-	-	15	-	-	16	-	-	-	-	-	18	-	-	17	-	-	16	-	-
12	-	-	-	16	-	-	-	-	-	16	-	-	18	-	-	18	-	-	16	-	-	16	-	-
13	37	-	-	-	-	-	44	-	-	44	-	-	27	-	-	41	-	-	44	-	-	43	-	-
14	27	-	-	35	-	-	23	-	-	24	-	-	52	-	-	38	-	-	34	-	-	33	-	-
15	-	-	-	21	-	-	-	-	-	-	-	-	-	-	-				-	-	-	-	-	-
16	-	-	-	-	-	-	-	-	-	-	-	-	-	-	-	-	-	-	-	-	-	-	-	-
17	-	-	-	-	-	-	-	-	-	-	-	-	-	-	-	-	-	-	-	-	-	-	-	-
18	-	-	-	-	-	-	-	-	-	-	-	-	-	-	-	-	-	-	-	-	-	-	-	-
19	-	-	-	-	-	-	-	-	-	-	-	-	-	-	-	-	-	-	-	-	-	-	-	-
20	-	-	-	-	-	-	-	-	-	-	-	-	-	-	-	-	-	-	-	-	-	-	-	-
21	-	-	-	-	-	-	-	-	-	10	-	-	-	-	-	-	-	-	-	-	-	-	-	-
22	12	-	-	-	-	-	10	-	-	-	-	-	12	-	-	10	-	-	-	-	-	-	-	-
23	13	-	-	14	-	-	19	-	-	16	-	-	24	-	-	27	-	-	25	-	-	35	-	-
24	-	-	-	-	-	-	-	-	-	-	-	-	-	-	-	-	-	-	-	-	-	-	-	-
25	-	-	-	-	-	-	-	-	-	-	-	-	-	-	-	-	-	-	-	-	-	-	-	-
26	10	-	-	-	-	-	-	-	-	-	-	-	-	-	-	-	-	-	-	-	-	-	-	-
27	28	-	-	24	-	-	20	-	-	19	-	-	33	-	-	23	-	-	35	-	-	25	-	-
28	13	-	-	12	-	-	-	-	-	11	-	-	15	-	-	12	-	-	-	-	-	14	-	-
29	12	-	-	-	-	-	-	-	-	-	-	-	-	-	-	-	-	-	-	-	-	-	-	-
30	-	-	-	-	-	-	-	-	-	-	-	-	-	-	-				-	-	-	-	-	-
31				-	-	-	-	-	-	-	-	-	-	-	-	-	-	-	-	-	-			
32	-	-	-				-	-	-	-	-	-	-	-	-	-	-	-	-	-	-			
33	-	-	-	-	-	-	-	-	-	-	-	-				-	-	-	-	-	-	-	-	-
34	-	-	-	-	-	-	14	-	-	-	-	-	10	-	-	11	-	-	12	-	-	-	-	-
35	-	-	-	-	-	-	-	-	-	-	-	-	-	-	-	-	-	-	-	-	-	-	-	-
36	-	-	-																			-	-	-
37	-	-	-	-	-	-	-	-	-	-	-	-				-	-	-	-	-	-	-	-	-
38	-	-	-	-	-	-	-	-	-	-	-	-	-	-	-	-	-	-	-	-	-	-	-	-
39	-	-	-	-	-	-	-	-	-	-	-	-	-	-	-	-	-	-	-	-	-			
40	15	-	-	17	-	-	-	-	-	12	-	-	13	-	-	30	-	-	25	-	-	14	-	-
41	-	-	-	-	-	-	-	-	-	-	-	-	-	-	-	-	-	-	-	-	-	-	-	-
42	-	-	-	-	-	-	-	-	-	-	-	-	-	-	-	-	-	-	-	-	-	-	-	-
43	11	-	-	-	-	-	-	-	-	-	-	-	-	-	-	-	-	-	-	-	-			
44							-	-	-	-	-	-	-	-	-	-	-	-	-	-	-	-	-	-
45	-	-	-	-	-	-	-	-	-	-	-	-	-	-	-	-	-	-	-	-	-	-	-	-
46	-	-	-	-	-	-	-	-	-	-	-	-	-	-	-	-	-	-	-	-	-	-	-	-
47	-	-	-	-	-	-	-	-	-	-	-	-	10	-	-	-	-	-	11	-	-	-	-	-

“-” indicates the value of <10.

**Table 4:** Coronary artery and aortic bypass grafting with >two anastomoses using a heart–lung machine (K5522): Case number and outcomes of postoperative in-hospital mortality.

Fiscal year	2011			2012			2013			2014			2015			2016			2017			2018		
Prefecture	Case	Outcome		Case	Outcome		Case	Outcome		Case	Outcome		Case	Outcome		Case	Outcome		Case	Outcome		Case	Outcome	
01	248	12	4.8%	280	16	5.7%	271	14	5.2%	354	22	6.2%	313	15	4.8%	338	25	7.4%	322	17	5.3%	338	20	5.9%
02	31	-	-	37	-	-	35	-	-	34	-	-	44	-	-	37	-	-	49	-	-	39	-	-
03	20	-	-	20	-	-	26	-	-	23	-	-	15	-	-	20	-	-	25	-	-	32	-	-
04	144	-	-	165	-	-	142	-	-	159	-	-	155	-	-	175	-	-	187	-	-	167	-	-
05	19	-	-	20	-	-	26	-	-	32	-	-	33	-	-	36	-	-	31	-	-	30	-	-
06	54	-	-	60	-	-	66	-	-	46	-	-	50	-	-	51	-	-	61	-	-	71	-	-
07	48	-	-	46	-	-	63	-	-	62	-	-	59	-	-	68	-	-	73	-	-	56	-	-
08	86	-	-	87	-	-	84	-	-	78	-	-	68	-	-	95	-	-	118	-	-	97	-	-
09	149	-	-	130	-	-	155	-	-	134	-	-	165	-	-	156	-	-	147	-	-	120	-	-
10	137	-	-	145	-	-	109	-	-	110	-	-	93	-	-	96	-	-	104	-	-	133	-	-
11	123	-	-	151	-	-	141	10	7.1%	161	-	-	176	12	6.8%	186	16	8.6%	198	17	8.6%	239	17	7.1%
12	250	-	-	316	15	4.7%	290	-	-	244	-	-	249	13	5.2%	272	19	7.0%	272	19	7.0%	256	10	3.9%
13	720	39	5.4%	731	37	5.1%	676	40	5.9%	702	37	5.3%	644	57	8.9%	720	36	5.0%	744	30	4.0%	665	26	3.9%
14	424	15	3.5%	471	20	4.2%	480	12	2.5%	420	21	5.0%	444	19	4.3%	375	17	4.5%	417	20	4.8%	380	21	5.5%
15	83	-	-	103	-	-	81	-	-	76	-	-	63	-	-	49	-	-	43	-	-	55	-	-
16	49	-	-	48	-	-	44	-	-	38	-	-	45	-	-	56	-	-	50	-	-	36	-	-
17	63	-	-	64	-	-	53	-	-	70	-	-	75	-	-	66	-	-	51	-	-	51	-	-
18	83	-	-	71	-	-	74	-	-	67	-	-	58	-	-	76	-	-	62	-	-	42	-	-
19	77	-	-	60	-	-	63	-	-	60	-	-	67	-	-	69	-	-	68	-	-	77	-	-
20	114	-	-	123	11	8.9%	114	-	-	113	-	-	136	-	-	118	-	-	111	-	-	103	-	-
21	36	-	-	52	-	-	59	-	-	80	-	-	69	-	-	79	-	-	90	-	-	96	-	-
22	152	-	-	190	10	5.3%	185	11	5.9%	168	-	-	203	-	-	173	-	-	171	-	-	169	-	-
23	405	19	4.7%	449	16	3.6%	390	11	2.8%	501	24	4.8%	502	21	4.2%	578	21	3.6%	564	17	3.0%	561	17	3.0%
24	60	-	-	74	-	-	59	11	18.6%	35	-	-	55	-	-	51	-	-	89	-	-	78	-	-
25	30	-	-	47	-	-	34	-	-	35	-	-	31	-	-	40	-	-	47	-	-	29	-	-
26	138	-	-	173	11	6.4%	175	-	-	137	-	-	111	12	10.8%	126	-	-	125	-	-	108	-	-
27	584	23	3.9%	632	19	3.0%	590	26	4.4%	566	33	5.8%	644	28	4.3%	654	31	4.7%	628	22	3.5%	683	26	3.8%
28	319	13	4.1%	335	13	3.9%	341	-	-	370	18	4.9%	287	14	4.9%	291	16	5.5%	289	15	5.2%	267	19	7.1%
29	193	10	5.2%	199	-	-	176	-	-	155	-	-	130	-	-	120	-	-	122	-	-	118	-	-
30	97	-	-	97	-	-	88	-	-	113	-	-	75	-	-	86	-	-	69	-	-	68	-	-
31	51	-	-	55	-	-	59	-	-	45	-	-	57	-	-	58	-	-	60	-	-	66	-	-
32	28	-	-	28	-	-	31	-	-	38	-	-	50	-	-	54	-	-	41	-	-	34	-	-
33	133	-	-	158	-	-	192	-	-	148	-	-	146	-	-	150	-	-	148	-	-	128	-	-
34	84	-	-	94	-	-	91	-	-	168	10	6.0%	203	-	-	142	-	-	131	-	-	130	-	-
35	65	-	-	63	-	-	43	-	-	55	-	-	49	-	-	76	-	-	75	-	-	60	-	-
36	39	-	-	27	-	-	21	-	-	18	-	-	20	-	-	26	-	-	24	-	-	22	-	-
37	23	-	-	41	-	-	45	-	-	56	-	-	55	-	-	48	-	-	34	-	-	41	-	-
38	28	-	-	28	-	-	30	-	-	54	-	-	37	-	-	67	-	-	71	-	-	51	-	-
39	76	-	-	57	-	-	80	-	-	84	-	-	95	-	-	101	-	-	86	-	-	72	-	-
40	269	13	4.8%	254	-	-	259	10	3.9%	307	18	5.9%	300	11	3.7%	307	-	-	398	15	3.8%	355	16	4.5%
41	41	-	-	36	-	-	29	-	-	31	-	-	36	-	-	52	-	-	49	-	-	42	-	-
42	161	-	-	147	-	-	168	-	-	166	-	-	136	-	-	115	-	-	139	-	-	136	-	-
43	123	-	-	143	-	-	153	-	-	140	-	-	159	-	-	128	-	-	97	-	-	97	-	-
44	24	-	-	28	-	-	21	-	-	22	-	-	32	-	-	32	-	-	32	-	-	30	-	-
45	84	-	-	83	-	-	88	-	-	100	-	-	93	-	-	79	-	-	76	-	-	94	-	-
46	109	-	-	154	-	-	129	-	-	115	-	-	144	-	-	137	-	-	137	10	7.3%	68	-	-
47	59	-	-	58	-	-	53	-	-	84	-	-	92	-	-	87	-	-	91	-	-	99	-	-

“-” indicates the value of <10.

**Table 5:** Coronary artery and aortic bypass grafting with one anastomosis without a heart–lung machine (K552-21): Case number and outcomes of postoperative in-hospital mortality.

Fiscal year	2011			2012			2013			2014			2015			2016			2017			2018		
Prefecture	Case	Outcome		Case	Outcome		Case	Outcome		Case	Outcome		Case	Outcome		Case	Outcome		Case	Outcome		Case	Outcome	
01	46	-	-	60	-	-	60	-	-	51	-	-	42	-	-	84	-	-	83	-	-	52	-	-
02	15	-	-	13	-	-	21	-	-	25	-	-	11	-	-	15	-	-	13	-	-	-	-	-
03	10	-	-	15	-	-	-	-	-	-	-	-	13	-	-	-	-	-	16	-	-	14	-	-
04	13	-	-	11	-	-	13	-	-	11	-	-	11	-	-	14	-	-	11	-	-	18	-	-
05	14	-	-	-	-	-	-	-	-	-	-	-	-	-	-	-	-	-	-	-	-	-	-	-
06	20	-	-	14	-	-	-	-	-	12	-	-	12	-	-	-	-	-	-	-	-	12	-	-
07	-	-	-	13	-	-	-	-	-	-	-	-	10	-	-	-	-	-	-	-	-	-	-	-
08	-	-	-	-	-	-	15	-	-	13	-	-	-	-	-	15	-	-	12	-	-	-	-	-
09	-	-	-	-	-	-	-	-	-	-	-	-	-	-	-	-	-	-	-	-	-	-	-	-
10	19	-	-	18	-	-	14	-	-	13	-	-	10	-	-	15	-	-	-	-	-	-	-	-
11	20	-	-	19	-	-	19	-	-	26	-	-	31	-	-	22	-	-	23	-	-	29	-	-
12	33	-	-	42	-	-	24	-	-	25	-	-	34	-	-	36	-	-	60	-	-	79	-	-
13	68	-	-	100	-	-	90	-	-	104	-	-	102	-	-	73	-	-	95	-	-	107	-	-
14	59	-	-	52	-	-	51	-	-	59	-	-	52	-	-	66	-	-	73	-	-	78	-	-
15	-	-	-	-	-	-	-	-	-	-	-	-	-	-	-	-	-	-	-	-	-	-	-	-
16	-	-	-	-	-	-	-	-	-	-	-	-	-	-	-	10	-	-	-	-	-	-	-	-
17	-	-	-	10	-	-	-	-	-	-	-	-	-	-	-	-	-	-	-	-	-	-	-	-
18	-	-	-	-	-	-	-	-	-	-	-	-	-	-	-	-	-	-	-	-	-	-	-	-
19	-	-	-	-	-	-				-	-	-	-	-	-	-	-	-				-	-	-
20	20	-	-	29	-	-	25	-	-	24	-	-	19	-	-	17	-	-	25	-	-	24	-	-
21	17	-	-	10	-	-	14	-	-	-	-	-	14	-	-	16	-	-	-	-	-	20	-	-
22	11	-	-	13	-	-	30	-	-	29	-	-	32	-	-	30	-	-	26	-	-	22	-	-
23	66	-	-	60	-	-	76	-	-	54	-	-	72	-	-	70	-	-	59	-	-	46	-	-
24	-	-	-	-	-	-	-	-	-	-	-	-	14	-	-	-	-	-	11	-	-	15	-	-
25	-	-	-	-	-	-	-	-	-	-	-	-	12	-	-	-	-	-	-	-	-	-	-	-
26	12	-	-	18	-	-	13	-	-	10	-	-	-	-	-	13	-	-	14	-	-	-	-	-
27	38	-	-	66	-	-	42	-	-	39	-	-	46	-	-	36	-	-	42	-	-	40	-	-
28	15	-	-	21	-	-	20	-	-	34	-	-	19	-	-	23	-	-	18	-	-	31	-	-
29	-	-	-	-	-	-	-	-	-	-	-	-	-	-	-	-	-	-	-	-	-	-	-	-
30	-	-	-	-	-	-	-	-	-	-	-	-	-	-	-	12	-	-	-	-	-	-	-	-
31	-	-	-	-	-	-	-	-	-	-	-	-	-	-	-	-	-	-	-	-	-	-	-	-
32	-	-	-	-	-	-	-	-	-	-	-	-	-	-	-	-	-	-	-	-	-	10	-	-
33	13	-	-	18	-	-	13	-	-	19	-	-	21	-	-	22	-	-	11	-	-	14	-	-
34	18	-	-	20	-	-	18	-	-	14	-	-	15	-	-	18	-	-	16	-	-	16	-	-
35	-	-	-	18	-	-	14	-	-	20	-	-	10	-	-	18	-	-	-	-	-	-	-	-
36	-	-	-	-	-	-	-	-	-				-	-	-	-	-	-	-	-	-	-	-	-
37	-	-	-	13	-	-	-	-	-	11	-	-	-	-	-	-	-	-	-	-	-	-	-	-
38	-	-	-	10	-	-	-	-	-	11	-	-	14	-	-	-	-	-	-	-	-	11	-	-
39	-	-	-				-	-	-	-	-	-	-	-	-	-	-	-	-	-	-	-	-	-
40	37	-	-	21	-	-	20	-	-	19	-	-	30	-	-	18	-	-	18	-	-	14	-	-
41	-	-	-	-	-	-	-	-	-	-	-	-				-	-	-	-	-	-	-	-	-
42	12	-	-	11	-	-	-	-	-	-	-	-	-	-	-	-	-	-	-	-	-	-	-	-
43	-	-	-	13	-	-	-	-	-	11	-	-	-	-	-	-	-	-	-	-	-	-	-	-
44	-	-	-	-	-	-	-	-	-	-	-	-	-	-	-	-	-	-	14	-	-	19	-	-
45				-	-	-	-	-	-	-	-	-	-	-	-	-	-	-	-	-	-	-	-	-
46	-	-	-	-	-	-	-	-	-	-	-	-	15	-	-	-	-	-	-	-	-	-	-	-
47	-	-	-	12	-	-	16	-	-	19	-	-	13	-	-	11	-	-	12	-	-	-	-	-

“-” indicates the value of <10.

**Table 6:** Coronary artery and aortic bypass grafting with >two anastomoses without a heart–lung machine (K552-22): Case number and outcomes of postoperative in-hospital mortality.

Fiscal year	2011			2012			2013			2014			2015			2016			2017			2018		
Prefecture	Case	Outcome		Case	Outcome		Case	Outcome		Case	Outcome		Case	Outcome		Case	Outcome		Case	Outcome		Case	Outcome	
01	414	10	2.4%	477	-	-	451	13	2.9%	469	-	-	562	-	-	528	-	-	545	-	-	471	13	2.8%
02	135	-	-	109	-	-	115	-	-	82	-	-	121	-	-	89	-	-	94	-	-	107	-	-
03	95	-	-	80	-	-	68	-	-	80	-	-	54	-	-	73	-	-	85	-	-	99	-	-
04	49	-	-	62	-	-	38	-	-	31	-	-	57	-	-	57	-	-	45	-	-	42	-	-
05	29	-	-	29	-	-	19	-	-	15	-	-	16	-	-	-	-	-	10	-	-	11	-	-
06	28	-	-	38	-	-	45	-	-	55	-	-	68	-	-	37	-	-	42	-	-	58	-	-
07	71	-	-	76	-	-	68	-	-	75	-	-	80	-	-	88	-	-	53	-	-	67	-	-
08	103	-	-	94	-	-	103	-	-	94	-	-	105	-	-	115	-	-	96	-	-	121	-	-
09	20	-	-	18	-	-	34	-	-	26	-	-	12	-	-	20	-	-	24	-	-	19	-	-
10	52	-	-	78	-	-	97	-	-	66	-	-	72	-	-	43	-	-	34	-	-	33	-	-
11	361	-	-	354	-	-	351	-	-	312	-	-	343	-	-	364	-	-	350	-	-	361	-	-
12	183	-	-	215	-	-	272	-	-	359	-	-	363	-	-	379	-	-	345	-	-	366	-	-
13	1024	14	1.4%	1178	10	0.8%	1148	21	1.8%	1168	13	1.1%	1144	15	1.3%	1048	14	1.3%	1083	15	1.4%	1083	-	-
14	339	-	-	331	-	-	318	-	-	391	-	-	475	-	-	503	10	2.0%	425	-	-	424	-	-
15	84	-	-	99	-	-	82	-	-	115	-	-	90	-	-	67	-	-	70	-	-	49	-	-
16	88	-	-	79	-	-	94	-	-	102	-	-	110	-	-	83	-	-	63	-	-	92	-	-
17	84	-	-	85	-	-	75	-	-	44	-	-	70	-	-	89	-	-	100	-	-	98	-	-
18	29	-	-	38	-	-	46	-	-	28	-	-	48	-	-	30	-	-				38	-	-
19										-	-	-	-	-	-	-	-	-	37	-	-	-	-	-
20	122	-	-	129	-	-	137	-	-	132	-	-	134	-	-	123	-	-	99	-	-	113	-	-
21	40	-	-	70	-	-	42	-	-	43	-	-	86	-	-	117	-	-	119	-	-	137	-	-
22	218	-	-	256	-	-	236	-	-	235	-	-	219	10	4.6%	223	-	-	218	-	-	226	-	-
23	466	-	-	512	-	-	547	-	-	496	-	-	490	-	-	495	-	-	502	-	-	518	11	2.1%
24	151	-	-	166	-	-	154	-	-	166	-	-	143	-	-	127	-	-	116	-	-	126	-	-
25	139	-	-	136	-	-	130	-	-	159	-	-	131	-	-	125	-	-	129	-	-	116	-	-
26	154	-	-	156	-	-	129	-	-	120	-	-	144	-	-	127	-	-	133	-	-	140	-	-
27	443	-	-	492	-	-	507	10	2.0%	453	10	2.2%	478	-	-	425	-	-	421	-	-	381	-	-
28	179	-	-	233	-	-	255	-	-	250	-	-	273	-	-	202	-	-	187	-	-	236	-	-
29	34	-	-	17	-	-	40	-	-	48	-	-	50	-	-	61	-	-	57	-	-	60	-	-
30	74	-	-	98	-	-	134	-	-	110	-	-	97	-	-	95	-	-	102	-	-	103	-	-
31	21	-	-	39	-	-	17	-	-	24	-	-	21	-	-	16	-	-	16	-	-	12	-	-
32	23	-	-	28	-	-	31	-	-	34	-	-	32	-	-	22	-	-	19	-	-	28	-	-
33	145	-	-	170	-	-	182	-	-	184	-	-	128	-	-	122	-	-	132	-	-	124	-	-
34	168	-	-	148	-	-	135	-	-	103	-	-	63	-	-	111	-	-	83	-	-	76	-	-
35	102	-	-	107	-	-	120	-	-	148	-	-	98	-	-	110	-	-	124	-	-	84	-	-
36	88	-	-	80	-	-	64	-	-	64	-	-	62	-	-	58	-	-	54	-	-	50	-	-
37	79	-	-	72	-	-	69	-	-	73	-	-	46	-	-	43	-	-	65	-	-	45	-	-
38	110	-	-	112	-	-	100	-	-	87	-	-	79	-	-	84	-	-	93	-	-	91	-	-
39	79	-	-	64	-	-	43	-	-	57	-	-	49	-	-	32	-	-	31	-	-	35	-	-
40	392	-	-	432	-	-	405	-	-	345	-	-	316	-	-	230	-	-	253	-	-	226	-	-
41	54	-	-	66	-	-	78	-	-	51	-	-	59	-	-	55	-	-	38	-	-	23	-	-
42	18	-	-	57	-	-	25	-	-	40	-	-	63	-	-	58	-	-	50	-	-	53	-	-
43	86	-	-	53	-	-	72	-	-	71	-	-	87	-	-	86	-	-	80	-	-	78	-	-
44	72	-	-	83	-	-	111	-	-	126	-	-	118	-	-	104	-	-	122	-	-	109	-	-
45	54	-	-	94	-	-	80	-	-	73	-	-	75	-	-	48	-	-	45	-	-	35	-	-
46	68	-	-	86	-	-	97	-	-	149	-	-	104	-	-	108	-	-	123	-	-	165	-	-
47	170	-	-	170	-	-	123	-	-	112	-	-	118	-	-	110	-	-	137	-	-	119	-	-

“-” indicates the value of <10.

### 3.4. Tracheotomy (K386)

**Fig 4:**
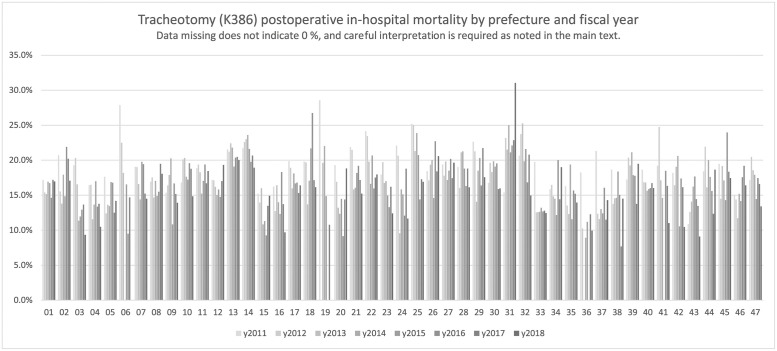
Tracheotomy (K386). Tracheotomy (K386) postoperative in-hospital mortality by prefecture and fiscal year.

Fig 4 depicts the postoperative in-hospital mortality, followed by Table 7 shows the results of tracheotomy cases. Data shown in Table 7 included relatively high numerical results for both the denominator and numerator; in several regions, comparisons in terms of temporal changes and regional differences could be made. Although the tabulation was an analysis at the prefectural level, which indicates that the collections include a broad range of data from each region, clear regional differences were depicted in the index values; thus, the implications should be carefully interpreted as presented in the Discussion. The temporal changes do not appear to be uniform among regions, although the results for all regions combined showed a decreasing trend over time.

**Table 7:** Tracheotomy (K386): Case number and outcomes of postoperative in-hospital mortality.

Fiscal year	2011			2012			2013			2014			2015			2016			2017			2018		
Prefecture	Case	Outcome		Case	Outcome		Case	Outcome		Case	Outcome		Case	Outcome		Case	Outcome		Case	Outcome		Case	Outcome	
01	862	250	29.0%	839	246	29.3%	852	264	31.0%	786	224	28.5%	903	295	32.7%	779	211	27.1%	820	243	29.6%	837	243	29.0%
02	232	69	29.7%	219	57	26.0%	203	44	21.7%	212	59	27.8%	229	63	27.5%	210	75	35.7%	203	62	30.5%	211	58	27.5%
03	140	47	33.6%	133	46	34.6%	133	41	30.8%	141	31	22.0%	117	35	29.9%	116	32	27.6%	117	33	28.2%	118	29	24.6%
04	213	56	26.3%	194	53	27.3%	199	48	24.1%	205	47	22.9%	200	50	25.0%	232	52	22.4%	196	43	21.9%	200	43	21.5%
05	170	62	36.5%	129	32	24.8%	154	50	32.5%	134	35	26.1%	160	50	31.3%	149	48	32.2%	160	50	31.3%	155	41	26.5%
06	122	57	46.7%	120	44	36.7%	99	36	36.4%	115	28	24.3%	121	33	27.3%	116	27	23.3%	109	34	31.2%	105	26	24.8%
07	299	116	38.8%	268	103	38.4%	217	70	32.3%	285	94	33.0%	248	91	36.7%	247	81	32.8%	276	81	29.3%	221	73	33.0%
08	313	105	33.5%	262	107	40.8%	266	83	31.2%	264	86	32.6%	289	94	32.5%	297	95	32.0%	277	95	34.3%	310	98	31.6%
09	241	67	27.8%	250	74	29.6%	229	69	30.1%	247	76	30.8%	277	57	20.6%	258	60	23.3%	244	57	23.4%	237	54	22.8%
10	214	73	34.1%	204	81	39.7%	197	69	35.0%	187	60	32.1%	238	91	38.2%	276	108	39.1%	197	69	35.0%	209	61	29.2%
11	667	224	33.6%	691	232	33.6%	624	198	31.7%	631	184	29.2%	680	199	29.3%	727	235	32.3%	737	200	27.1%	775	238	30.7%
12	640	196	30.6%	613	195	31.8%	599	177	29.5%	580	156	26.9%	683	196	28.7%	664	195	29.4%	670	197	29.4%	688	214	31.1%
13	1917	620	32.3%	1815	560	30.9%	1740	558	32.1%	1858	557	30.0%	1921	550	28.6%	1764	496	28.1%	1784	510	28.6%	1698	480	28.3%
14	995	358	36.0%	863	314	36.4%	935	324	34.7%	970	349	36.0%	1004	304	30.3%	982	292	29.7%	1036	328	31.7%	1014	267	26.3%
15	184	57	31.0%	172	57	33.1%	175	46	26.3%	157	40	25.5%	168	44	26.2%	162	49	30.2%	163	49	30.1%	181	50	27.6%
16	154	49	31.8%	157	46	29.3%	134	35	26.1%	150	43	28.7%	138	36	26.1%	153	50	32.7%	153	39	25.5%	124	34	27.4%
17	136	50	36.8%	111	40	36.0%	119	42	35.3%	138	49	35.5%	120	50	41.7%	125	44	35.2%	111	46	41.4%	128	50	39.1%
18	101	37	36.6%	61	25	41.0%	95	32	33.7%	82	28	34.1%	83	32	38.6%	86	37	43.0%	70	21	30.0%	62	22	35.5%
19	56	22	39.3%	57	15	26.3%	51	13	25.5%	59	21	35.6%	74	27	36.5%	64	15	23.4%	102	25	24.5%	76	20	26.3%
20	228	79	34.6%	213	64	30.0%	189	64	33.9%	162	45	27.8%	166	47	28.3%	153	36	23.5%	167	52	31.1%	154	57	37.0%
21	215	93	43.3%	177	64	36.2%	215	71	33.0%	193	63	32.6%	187	65	34.8%	177	62	35.0%	198	68	34.3%	184	58	31.5%
22	393	142	36.1%	371	144	38.8%	379	139	36.7%	343	107	31.2%	329	108	32.8%	301	82	27.2%	314	89	28.3%	334	99	29.6%
23	596	201	33.7%	604	197	32.6%	645	176	27.3%	653	194	29.7%	650	182	28.0%	669	150	22.4%	685	204	29.8%	644	156	24.2%
24	163	59	36.2%	160	54	33.8%	115	25	21.7%	158	47	29.7%	172	52	30.2%	174	52	29.9%	165	50	30.3%	163	40	24.5%
25	131	48	36.6%	144	59	41.0%	122	43	35.2%	134	53	39.6%	135	52	38.5%	139	43	30.9%	133	50	37.6%	142	51	35.9%
26	331	122	36.9%	362	112	30.9%	310	103	33.2%	335	108	32.2%	336	99	29.5%	361	129	35.7%	337	107	31.8%	345	117	33.9%
27	1181	393	33.3%	1141	375	32.9%	1182	381	32.2%	1135	337	29.7%	1237	376	30.4%	1234	396	32.1%	1222	362	29.6%	1293	383	29.6%
28	683	219	32.1%	674	201	29.8%	597	198	33.2%	602	200	33.2%	675	214	31.7%	674	222	32.9%	670	215	32.1%	633	176	27.8%
29	243	93	38.3%	207	66	31.9%	199	54	27.1%	227	63	27.8%	202	69	34.2%	244	70	28.7%	221	66	29.9%	222	76	34.2%
30	167	46	27.5%	153	49	32.0%	142	40	28.2%	121	40	33.1%	131	38	29.0%	133	39	29.3%	126	41	32.5%	119	32	26.9%
31	91	29	31.9%	95	41	43.2%	93	34	36.6%	84	32	38.1%	90	31	34.4%	86	24	27.9%	105	40	38.1%	116	56	48.3%
32	121	40	33.1%	118	57	48.3%	103	41	39.8%	121	52	43.0%	125	66	52.8%	101	50	49.5%	101	43	42.6%	87	35	40.2%
33	309	106	34.3%	287	80	27.9%	262	83	31.7%	277	82	29.6%	280	88	31.4%	292	74	25.3%	297	85	28.6%	305	82	26.9%
34	322	82	25.5%	334	110	32.9%	310	88	28.4%	290	83	28.6%	304	81	26.6%	305	100	32.8%	333	107	32.1%	305	101	33.1%
35	221	72	32.6%	207	56	27.1%	195	47	24.1%	227	89	39.2%	190	56	29.5%	249	81	32.5%	204	78	38.2%	215	69	32.1%
36	115	26	22.6%	127	20	15.7%	120	18	15.0%	112	23	20.5%	125	32	25.6%	98	14	14.3%	106	24	22.6%	121	30	24.8%
37	169	60	35.5%	170	38	22.4%	138	36	26.1%	146	35	24.0%	153	39	25.5%	137	45	32.8%	113	33	29.2%	126	30	23.8%
38	134	43	32.1%	138	43	31.2%	117	43	36.8%	123	32	26.0%	136	53	39.0%	133	42	31.6%	143	38	26.6%	138	30	21.7%
39	174	60	34.5%	157	56	35.7%	135	47	34.8%	142	55	38.7%	168	64	38.1%	135	45	33.3%	138	40	29.0%	118	43	36.4%
40	826	263	31.8%	807	257	31.8%	720	221	30.7%	755	219	29.0%	794	216	27.2%	799	213	26.7%	843	262	31.1%	817	227	27.8%
41	104	34	32.7%	101	35	34.7%	111	34	30.6%	96	26	27.1%	105	26	24.8%	119	38	31.9%	98	27	27.6%	109	25	22.9%
42	220	78	35.5%	201	58	28.9%	194	62	32.0%	170	55	32.4%	199	50	25.1%	190	59	31.1%	186	55	29.6%	172	42	24.4%
43	257	56	21.8%	214	48	22.4%	220	54	24.5%	228	59	25.9%	232	79	34.1%	201	51	25.4%	193	48	24.9%	209	39	18.7%
44	201	64	31.8%	187	65	34.8%	180	69	38.3%	200	77	38.5%	193	64	33.2%	186	64	34.4%	170	48	28.2%	177	67	37.9%
45	154	56	36.4%	145	48	33.1%	146	45	30.8%	152	45	29.6%	161	39	24.2%	146	54	37.0%	147	43	29.3%	149	41	27.5%
46	279	88	31.5%	264	94	35.6%	239	87	36.4%	204	69	33.8%	247	93	37.7%	239	91	38.1%	255	99	38.8%	262	96	36.6%
47	355	112	31.5%	342	107	31.3%	269	83	30.9%	268	78	29.1%	256	65	25.4%	321	90	28.0%	265	75	28.3%	276	68	24.6%

“-” indicates the value of <10.

## 4. Discussion

The present study analyzed surgical outcomes at hospitals throughout Japan according to regional differences and temporal changes using the highly comprehensive DPC database and provided thorough results. Despite the simplicity of the method and broad analysis at the prefectural level, regional differences were observed in all surgical fields. The analysis of the administrative data was useful because this report confirmed the advantage of the Japanese DPC, in which DPC data are accompanied by the creation of Form 1 that corresponded to the hospitalization summary.

The most important aspect in the results of this study was its comprehensiveness in managing data from numerous hospitals. Some reports have indicated that Japan has fewer perioperative complications than other countries [1, 2]; however, from a global perspective, it is of interest to determine whether such trends observed in those reports are the same in hospitals throughout Japan, including hospitals not included in the registry data or whether this is common trends across all surgical fields. Data available from the DPC database, which now accumulates data from numerous Japanese hospitals, facilitate the extensive analysis of actual conditions that may not always be collectible by a registry or other sources. The database enables the investigation of actual implementation of procedures in a manner that was not possible before. In addition, all surgeries were included in this analysis, although the interpretation of the results varied and caution should be exercised while inferring them. Complete results are presented as S1 Data.

In the current study, the results from only some selections from all fields were presented; however, in the complete results, regional differences and temporal changes could be easily observed to some extent. Moreover, negative results about records without noticeable differences or those that remained unchanged was important for review. Although it depends on the purpose of the future research or project, the availability of data in all fields, such as that presented in this study, is considered extremely valuable.

The present study has several limitations and important caveats. In terms of data, several missing values were noted. For example, if the number of cases was 300, 3% of the outcomes was only 9 cases; this value was not obtainable as an aggregate value. In this study the data was compared at a general level, i.e., the prefectural level for analyzing the data of each year, as the author considered it reasonable to obtain tangible results; however, the number of nulls encountered were more than expected. A wider geographic aggregation could have been used to resolve the issue; however, this approach was not used in this study as the author, instead, preferred to conduct comparisons at a more detailed smaller regional level than the prefecture level or at a specific time span. Another issue encountered during the analysis was that the surgical code for artificial head insertion (K0811) included the surgery for both the shoulder and hip. A separate analysis should be conducted by combining disease names or using different master codes from different DPC datasets. However, a simple detailed analysis will yield fewer figures that can be obtained or presented. Although the present study conducted a relatively coarse unit-level analysis, the results showed some trends and differences and can be useful for future studies for information, such as knowledge about the fields requiring a detailed analysis or regions from where data ought to be collected and analyzed more thoroughly.

In the present study, in-hospital mortality was selected as the outcome; moreover, postoperative 30-day in-hospital mortality was considered. However, the number of outcomes within 30 days was naturally smaller and could increase missing data. Furthermore, being a rule of data publication, it was necessary to avoid inference from other data with cells having <10 records. For example, if presenting both in-hospital mortality and postoperative 30-day in-hospital mortality, it could be partly possible to calculate a small number of in-hospital mortality after 31 days. Therefore, only one type of outcome was used in this study; however, balancing the presentation of useful data with privacy considerations was challenging.

The resulting unattained numerical frames were rather expected; however, slightly more missing data were generated. For example, in coronary artery bypass surgery, the author had assumed that a large number of outcomes could have appeared and that those actual comparisons would be possible in more regions. However, both the number of outcomes and the number of cases were small in some regions; therefore, several numerical results were unobtainable. To circumvent this, it was possible to combine four separate coronary artery bypass procedures into one analysis or to integrate several similar procedures. It would be more useful to present both the combined and fine-grained aggregate values; however, in principle, we must avoid using those aggregate values to calculate a small numerical result. Thus, the more detailed results were presented in this study.

Deeper analysis is required to interpret the results associated with the missing values. Missing data should not be assumed to be zero or low values. Specifically, for comparison using graphs, the missing values appear to be zero because they do not exist or may get neglected. The missing values should be carefully re-evaluated differently, such as by integrating several fields, reanalyzing them, or referring to other information.

Furthermore, mortality outcomes are not necessarily bad outcomes. Analyses that use postoperative death or survival as an outcome can be primarily those that equate them with the “successfulness of surgery or perioperative procedures” to some extent. For example, tracheostomies are often performed to continue the treatment of patients with respiratory disorders. In such cases, the result refers to whether death or survival can often be independent from surgical techniques and perioperative management. This can be true for several other surgeries. For example, a patient who would otherwise have died if a certain surgery, such as coronary artery bypass surgery, was not performed would die as a result of the procedure.

Nevertheless, comparisons of outcomes can and should be discussed from various perspectives. A perspective to consider is whether a surgery should have been performed even if there were “no” other possible treatments. Meanwhile, there may exist areas with inadequate medical care that cannot be implemented until the last resort. Therefore, regarding outcomes in quality of care, the background information of cases such as instances of case-mix must be considered. Such a detailed study interpretation requires a comprehensive analysis with a more elaborate protocol; however, it is important to first present the current status, such as in this study, and to keep the interpretation flexible.

Other minor details include normalizing the minor inconsistencies in the data. For example, the K-code may contain a 2-byte character “K” that is different from the original code or the data may be recorded with codes that do not exist. Therefore, the conversion and unification of these characters can be considered when using the dataset. Although “blood transfusion” was found in data that should not be reported as surgeries in the Form 1 of DPC data as per input rules, unlike pre-maintained databases such as registry databases, caution should be exercised while using unclean raw data. The present study requested data acquisition according to the standard established rules at the beginning of the study, and managing the data inconsistencies and small numerical cells as described above had to be planned. Subsequently, it is now possible to conduct the study using individual data, which should facilitate a more flexible analysis in the future.

## 5. Conclusions

The present study analyzed surgical outcomes at hospitals throughout Japan by region and over time using the highly comprehensive DPC database, provided comprehensive result data, and depicted regional differences and temporal changes.

## Supporting information

S1 Data All data obtained in this study. All data obtained and all results in this study.
(XLSX)
