# Supplementary material for: Postoperative mortality analysis on nationwide data from diagnosis procedure combination database in Japan
Source: PLoS One. 2023 Jun 8;18(6):e0286264. doi: 10.1371/journal.pone.0286264 (PMC10249857; doi:10.1371/journal.pone.0286264)
Supplement: Attachment — Submitted filename: Response_to_Reviewers3.docx [file pone.0286264.s002.docx]

Response to Reviewers

For Journal requirements:

*Please ensure that your manuscript meets PLOS ONE's style requirements, including those for file naming. The PLOS ONE style templates can be found at*

*https://journals.plos.org/plosone/s/file?id=wjVg/PLOSOne_formatting_sample_main_body.pdf and*

[*https://journals.plos.org/plosone/s/file?id=ba62/PLOSOne_formatting_sample_title_authors_affiliations.pdf*](https://journals.plos.org/plosone/s/file?id=ba62/PLOSOne_formatting_sample_title_authors_affiliations.pdf)

*Thank you for stating the following in the Acknowledgments Section of your manuscript:*

*“Funding: This study was supported by Japan Society for the Promotion of Science (grant number 20K18961). The funders had no role in study design, data collection and analysis, decision to publish, or preparation of the manuscript.”*

*We note that you have provided funding information that is currently declared in your Funding Statement. However, funding information should not appear in the Acknowledgments section or other areas of your manuscript. We will only publish funding information present in the Funding Statement section of the online submission form.*

*Please remove any funding-related text from the manuscript and let us know how you would like to update your Funding Statement. Currently, your Funding Statement reads as follows:*

*“This study was supported by Japan Society for the Promotion of Science (grant number 20K18961). The funders had no role in study design, data collection and analysis, decision to publish, or preparation of the manuscript.”*

*Please include your amended statements within your cover letter; we will change the online submission form on your behalf.*

Thank you for the notifications. I have removed that part from my manuscript, and I have no update for the funding information.

Please provide additional details regarding participant consent. In the ethics statement in the Methods and online submission information, please ensure that you have specified what type you obtained (for instance, written or verbal, and if verbal, how it was documented and witnessed). If your study included minors, state whether you obtained consent from parents or guardians. If the need for consent was waived by the ethics committee, please include this information.

Please include your full ethics statement in the ‘Methods’ section of your manuscript file. In your statement, please include the full name of the IRB or ethics committee who approved or waived your study, as well as whether or not you obtained informed written or verbal consent. If consent was waived for your study, please include this information in your statement as well.

Accordingly, I have added the ethics statement in the last paragraph of the Methods section as follows:

“The need for obtaining individual informed consent was waived for this study as that data were anonymized and provided by the MHLW in accordance with the “Strategy for the Revitalization of Japan” 2016 (approved by the Cabinet on June 2, 2016)”

For Additional Editor Comments:

Though it addresses an important issue, it is not clear how much the manuscript will interest international readers. As the Review 2 pointed out, the authors need to restructure the background and discussion sections, so as to make the manuscript more relevant not just in Japan but globally.

Thank you for your advice. I have revised the Background and Discussion sections for providing a global perspective.

For Reviewer #1:

Thank you for your careful review.

For Reviewer #2:

Thank you very much for your kind advice.

It is also recommended that the authors re-consider about presentation of results. The tables simply filled with numbers are not easy for readers to capture what the main results are. Presenting the results in graphic figures may be one option, with raw numbers as supplementary data.

I agree that the graphs help in clearly explaining the data. Considering that graphs were the main outputs of this study, I have revised the manuscript so that readers can understand the graphs easily.

I have rephrased the four results as shown in the following examples.

“Figure 1 depicts the postoperative in-hospital mortality of artificial head insertion for the shoulder or hip in orthopedic surgery, and Table 1 shows the detail results for those cases.”

The authors reported on graphical and temporal changes in mortality rates surgical procedures using nationwide database. As the results may be challenging for readers outside Japan to understand, this reviewer suggests that the authors describe their findings from a more general point of view.

Thank you for your advice. I have revised the Discussion section for providing a general perspective.

In the introduction section, the authors focused primarily on the National Clinical Database and diagnosis procedure combination database, which are both nationwide domestic database in Japan. However, it is recommended to begin with more general statements that emphasize the limitation not only of domestic datasets but also of global datasets and the unique contributions of the current analysis.

Thank you for your advice. I have revised the Introduction section for providing a more global perspective.

In regards to the material and methods section, it is less meaningful to include database-specific terms such as “K-code”, “-“, and “*-“ in the section. Instead, it is recommended to use more commonly understood terms.

Thank you for the advice.

It is true that K-code is unique to Japan, and foreign readers find it difficult to understand this code. However, since the actual data were coded using this system and the Supporting Information provided the original data, I have revised the text and stated that this code is unique to Japan. I have also emphasized on the importance of presenting this code in this report.

Regarding “-“ and “*-“, I set these symbols originally when I deleted the data to accommodate the minimum number. I have revised the text to explain the significance of these symbols clearly.

Additionally, it is important to state the reasons why values of < 10 are not analyzed (for the anonymization purposes?).

Yes, this rule was set by the government to enhance anonymity. I have added this information in the revised manuscript.

“Data that can be obtained as an aggregate has been limited by the rules of MHLW to a value of ≥10 in each cell for anonymity.”

In the methods section, it is not clear why the authors focused on four specific procedures (K0811, K1771, K5521-552-22, and K386) in the results section. The discussion section suggests that regional differences and temporal changes can be observed to some extent in the complete results, but this information is not provided in the current manuscript.

In Material and methods, I had described the selection criteria; I have revised the text describing the criteria to enhance clarity.

“Across the entire spectrum, each result has its own importance and requires interpretation, but this report selected the most distinctive ones for consideration. Although this selection was arbitrarily made by the author, the inclusion criteria were as follows: those with relatively high mortality rates, those with visible temporal changes or regional differences, those requiring interpretation, or those requiring caution in future data analysis.”

In the opinion of this reviewer, the easiest way to avoid inference from other data with cells having <10 records is not to split data into 47 prefectures but rather to split data into larger area such as Kanto, Kinki etc. Please clearly state the rationale to analyze the dataset at the prefectural level.

I agree with your advice. Originally, I wanted to look at the differences in a relatively more detailed regional units; for example, at the level of secondary medical regions or cities. However, I thought that this would reduce the number of nulls, so I planned to work on the prefectural level, which is one of the general units. I thought that it would be good grouping to some extent. The results showed fewer outcomes in particular and more nulls than I expected.

I revised the text to indicate this more clearly.

Additionally, I have mentioned in the last paragraph of the Discussion section that this report follows the standard rules. However, the rule has now changed, and it is now possible to conduct the study using individual data, which could help in performing a flexible analysis in the future, such as by changing the grouping to the proper levels.

“A wider geographic aggregation could have been used to resolve the issue; however, this approach was not used in this study as the author, instead, preferred to conduct comparisons at a more detailed smaller regional level than the prefecture level or at a specific time span.”
